# Supplementary figures and images for: A comparative study of the cryo-EM structures of Saccharomyces cerevisiae and human anaphase-promoting complex/cyclosome (APC/C)
Source: eLife. 2024 Oct 14;13:RP100821. doi: 10.7554/eLife.100821 (PMC11473103; doi:10.7554/eLife.100821)

Figure 1-figure supplement 1  
Source data 1

A

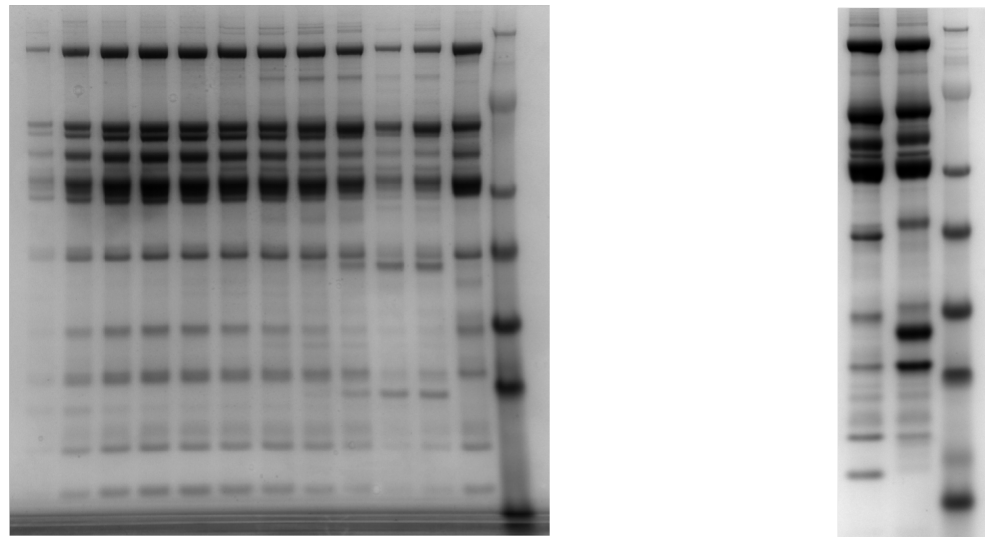

B

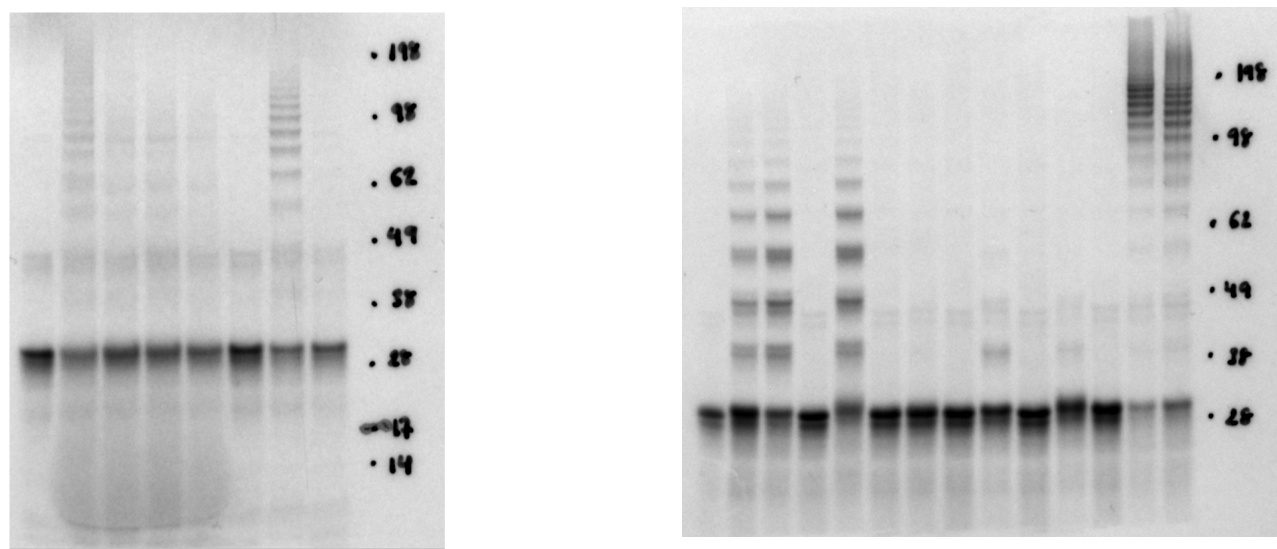

C

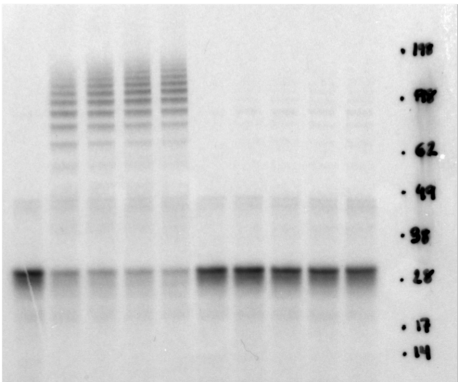

Supplement: Figure 1—figure supplement 1—source data 1. [file elife-100821-fig1-figsupp1-data1.zip › Source data 1.pdf]

Figure 1-figure supplement 1  
Source data 2

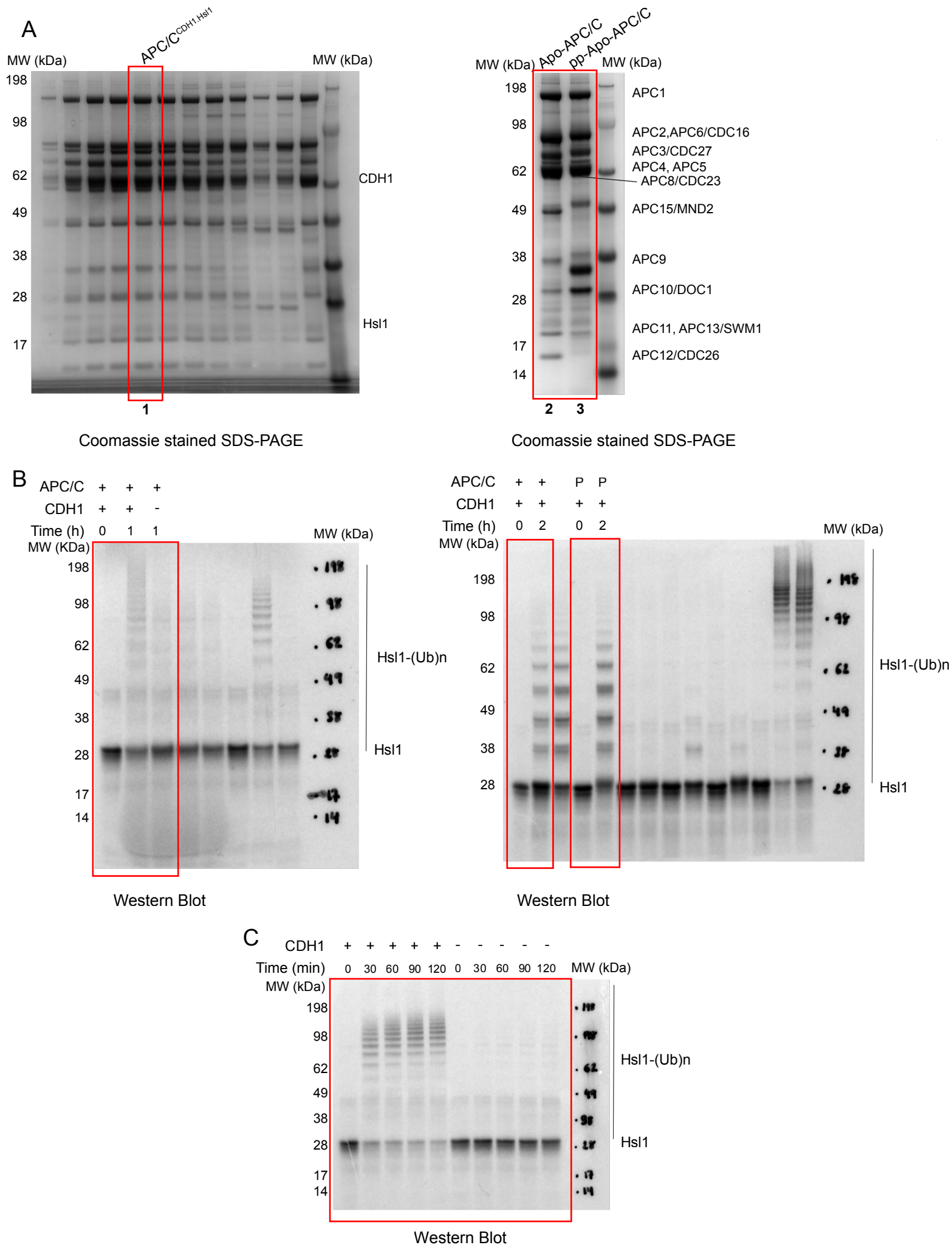

Supplement: Figure 1—figure supplement 1—source data 2. [file elife-100821-fig1-figsupp1-data2.zip › Source data 2.pdf]
