# Supplementary material for: A comparative study of the cryo-EM structures of Saccharomyces cerevisiae and human anaphase-promoting complex/cyclosome (APC/C)
Source: eLife. 2024 Oct 14;13:RP100821. doi: 10.7554/eLife.100821 (PMC11473103; doi:10.7554/eLife.100821)
Supplement: Supplementary file 1. — (a) Table of CDK Phosphorylation sites of S. cerevisiae APC/C. (b) Ordered and disordered regions of S. cerevisiae APC/C subunits [file elife-100821-supp1.docx]

**Supplementary File 1a. Summary of CDK phosphorylation sites of *in vitro* phosphorylated apo-APC/C identified by mass spectrometry**

| **Protein** | **Phospho-sites** | | | | | | | | | |
| --- | --- | --- | --- | --- | --- | --- | --- | --- | --- | --- |
| **APC1** | S131 | S133 | T232 | S234 | S235 | T236 | S247 | S250 | S254 | **S261** |
|  | **S267** | **T268** | **S269** | S274 | S285 | S292 | T295 | S303 | S305 | **S308** |
|  | **S310** | T312 | T323 | S329 | S335 | S336 | T339 | T341 | S343 | T346* |
|  | S349 | T363 | T365 | S444* | T499** | S500 | S528 | Y672 | S678 | S680 |
|  | T682 | S691 | T692 | S702 | S822 | S838 | S870 | S872 | S879 | **S884** |
|  | S897 | T992 | S1174 | **S1192** | S1193 | Y1206 | S1208 | **S1209** | S1418 | S1433 |
|  | S1436 | T1439 | T1442 | S1443 | T1447* | S1451 | T1459 | **S1462** | **S1469** | S1560 |
|  | S1588 | T1589* | S1647 | S1669 | T1681 | S1683 | S1688 | T1695 | T1701 | T1709 |
|  | S1728 | S1742 |  |  |  |  |  |  |  |  |
| **APC3** | T11 | S13 | S18 | S134 | S224 | **S225** | S228 | T229 | S230 | S232 |
|  | **S238** | S241 | S244 | T246 | T260 | **S265** | **S267*** | S270 | T271 | S272 |
|  | S274 | S275 | S278 | S285 | T292 | S293 | T296 | T304* | S312 | S316 |
|  | S323 | **S328*** | S330 | T335 | S337 | S338 | **T350** | **T351*** | S354 | T394 |
|  | T397 | S403 | S404 | T405 | T409 | T410 | S423 | T567 | S585 | S586 |
|  | T711 | T719 | T734 |  |  |  |  |  |  |  |
| **APC2** | **T81** | S82 | S87 | T268 | T269 | T270 | T426 | **S434*** | T562 | S572 |
|  | S706 | S708 | T735 | S737 | S742 | T752 | S760 | S768 | T777 | S781 |
|  | **S802** | S842 |  |  |  |  |  |  |  |  |
| **APC4** | Y8 | S143 | S446 | S459 | S553 | S554 | S563 | S578 | S588 | Y590 |
|  | S592 | T596 | Y598 | Y603 | T611 | S635 | S640 | S642 |  |  |
| **APC5** | S68 | S209 | T262 | S321 | S341 | S361 | T366 | S484 | T497 | S507 |
|  | S673 |  |  |  |  |  |  |  |  |  |
| **APC8** | S5 | S25 | T52 | **S54** | **S59**** | T94 | **S135** | T141 | T142 | S159 |
|  | S167 | S183 | S185 | S188 | T337 | S339 | S370 | S416 | T531 | S537 |
| **APC15** | S9 | **S51** | S71 | S87 | S101 | T127 | Y129 | S136 | T184 | T189 |
|  | S193 | S198 | S222 | Y223 | T268 | S269 | **S273** | **S293** | S300 | Y324 |
|  | S325 | T329 | S330 | Y331 | **T335*** | T346 | T351 | **S352** |  |  |
|  | | | | | | | | | | |

| **Protein** | **Phospho-sites** | | | | | | | | | |
| --- | --- | --- | --- | --- | --- | --- | --- | --- | --- | --- |
| **APC6** | **S44*** | **S46** | S50 | S54 | **T55** | S59* | S63 | S66 | T70 | S73 |
|  | T76 | T90 | T92 | **S95*** | Y97 | S99 | S103* | T115* | S120 | S123 |
|  | S127 | S129 | T131 | S143 | S145 | S148 | S152 | T153 | S156 | T157 |
|  | S170 | T209 | T211 | T212 | T214 | T215 | T216 | T217 | T218 | T220 |
|  | S225 | S228 | S305 | S326 | T331 | T332 | T336 | S338 | S344 | S360 |
|  | S438 | T447 | **S449** | S468 | T475 | S734 | Y736 | S742 | S743 | T757 |
|  | S763 | S770 | S774 | **S777** | S782 | **S788** | **S789** | T791 | **Y792** |  |
| **APC9** | S17 | T23** | T35 | T36** | T43 | S50 | T54 | T62 | Y67 | T81 |
|  | S83 | S94* | S109 | S159 | T183 | S185 | S205 | S210 | S248 | **S258** |
|  | S262 | S263 |  |  |  |  |  |  |  |  |
| **APC10** | S30 | T85 | T111 | S223 | T230 | T236 |  |  |  |  |
| **APC13** | T79 | S80 | S121 | T140 | T142 | S144 | T151 | S169 |  |  |
| **APC11** | S10 | S15 | S22 | T84* | T145 | T156 |  |  |  |  |
| **APC12** | T8 | **S12** | T17 | T37 | T57 | S58 | S79 | S96 | S100 | T101 |
|  | S103 | T105 | T109 | **S110** |  |  |  |  |  |  |
| The phosphorylation sites in **bold** are present in both complexes: phosphorylated apo-APC/C and the control unphosphorylated apo-APC/C. The sequence coverage is ≥77% for all the subunits.  * Consensus CDK site (S/T)P  ** Consensus CDK site (S/T)Px(K/R) | | | | | | | | | | |

**Supplementary File 1b. Ordered and disordered regions of APC/C^CDH1:Hsl1^ subunits**

| Subunit | Human  ortholog | Visible  N-term | Visible  C-term | Disordered regions | ^1^AF2 C-term helix | Protein length (N) | Domain/region 1 | Domain/region 2 | Domain/region 3 | Identity/similarity/gaps to human (%) |
| --- | --- | --- | --- | --- | --- | --- | --- | --- | --- | --- |
| APC1 | APC1 | 27 | 1746 | 134-141, 170-188, 224-366, 388-9, 676-691, 853-4, 873-893, 1189-1210, 1671-6, 1706-9 | - | 1748 | 1-534 (WD40) | 524-894,  1534-1748 (Mid) | 893-1533 (PC) | 19.9/35.2/32.0 |
| APC2 | APC2 | 4 | 746 | 69-91, 420-453, 473-484, 519-524, 545-566, 620-4 | - | 853 | 1-544 (cullin repeats) | 545-746 (CTD) | 746-853 (WHB) | 18.4/32.3/37.3 |
| APC3A/  CDC27 | APC3 | 23 | 755 | 132-142, 210-433 | 756 | 758 | TPR superhelix | - | - | 26.3/41.8/22.8 |
| APC3B/  CDC27 | APC3 | 20 | 755 | 132-140, 210-431 | 756 | 758 | TPR superhelix | - | - |  |
| APC4 | APC4 | 5 | 646 | 524-528 | - | 652 | 1-225, 477-652 (WD40) | 226-476 (4HB) | - | 16.0/29.3/42.0 |
| APC5 | APC5 | 3 | 675 | 261-275 | 684 | 685 | 1-297 (NTD) | 298-675 (TPR superhelix) | - | 16.7/31.5/33.3 |
| APC6A/  CDC16 | APC6 | 229 | 761 | 328-351 | 757 | 840 | TPR superhelix |  | - | 21.7/37.0/36.0 |
| APC6B/  CDC16 | APC6 | 229 | 758 | 327-351 | 757 | 840 | TPR superhelix | - | - |  |
| APC8A/  CDC23 | APC8 | 6 | 626 | 45-74 | 626 | 626 | TPR superhelix | - |  | 26.5/41.8/31.3 |
| APC8B/  CDC3 | APC8 | 4 | 626 | 47-73 | 626 | 626 | TPR superhelix | - | - |  |
| APC9 | APC16 | 89 | 259 | 125-133, 143-158, 181-190, 241-246 | - | 265 | Segment 1:  89-142 | Segment 2:  159-240 | Segment 3:  247-259 | 9.7/18.1/63.4 |
| APC10/  DOC1 | APC10 | 4 | 250 | 224-246 | - | 250 | 1-223 DOC | 247-250 (IR tail) | - | 22.5/38.0/31.4 |
| APC11 | APC11 | 1 | 130 | 21-36 | - | 165 | 1-36 (β-strand) | 37-130 (RING) | - | 27.3/33.9/49.1 |
| APC12A/CDC26 | APC12 | 1 | 35 | - | - | 124 | 1-12 (β-strand) | 13-35 (α-helix) | - | 18.1/29.7/48.6 |
| APC12B/CDC26 | APC12 | 1 | 37 | - | - | 124 | 1-12 (β-strand) | 13-35 (α-helix) | - |  |
| APC13/  SWM1 | APC13 | 2 | 166 | 46-77, 117-138 | - | 170 | - | - | - | 11.6/17.9/59.0 |
| APC15/  MND2 | APC15 | 2 | 140 | 21-63 | - | 368 | - | - | - | 10.2/15.6/68.5 |
| CDH1 | CDH1 | 52 | 566 | 76-89, 135-186 | - | 566 | 1-245 (NTD) | 246-546 (WD40) | 556-66 (IR tail) | 36.4/52.3/20.9 |
| None | APC7 | - | - | - | - | - | - | - | - | - |
|  | | | | | | | | | |  |
| ^2^Hsl1 | - | 831 | 836 | - | - | 667-782 | - | - | - | - |

^1^C-terminal residue of an AF2-predicted C-terminal α-helix.

^2^*S. cerevisiae* APC/C substrate
